# Efficacy and Safety of Deep Anterior Lamellar Keratoplasty *vs.* Penetrating Keratoplasty for Keratoconus: A Meta-Analysis

**DOI:** 10.1371/journal.pone.0113332

**Published:** 2015-01-29

**Authors:** Hao Liu, Yihui Chen, Peng Wang, Bing Li, Weifang Wang, Yan Su, Minjie Sheng

**Affiliations:** Department of Ophthalmology, Shanghai tenth people’s Hospital, Tongji University, Shanghai, 200072, China; Boston University School of Medicine, UNITED STATES

## Abstract

**Purpose:**

To evaluate difference in therapeutic outcomes between deep anterior lamellar keratoplasty (DALK) and penetrating keratoplasty (PKP) for the clinical treatment of keratoconus.

**Methods:**

A comprehensive search was conducted in Pubmed, EMBASE, Cochrane Library, and Web of science. Eligible studies should include at least one of the following factors: best corrected visual acuity (BCVA), postoperative spherical equivalent (SE), postoperative astigmatism and endothelial cell count (ECC), central corneal thickness (CCT), graft rejection and graft failure, of which BCVA, graft rejection and graft failure were used as the primary outcome measures, and postoperative SE, astigmatism, CCT and ECC as the secondary outcome measures. Given the lack of randomized clinical trials (RCTs), cohort studies and prospective studies were considered eligible.

**Results:**

Sixteen clinical trials involving 6625 eyes were included in this review, including 1185 eyes in DALK group, and 5440 eyes in PKP group. The outcomes were analyzed using Cochrane Review Manager (RevMan) version 5.0 software. The postoperative BCVA in DALK group was significantly better than that in PKP group (OR = 0.48; 95%CI 0.39 to 0.60; p<0.001). There were fewer cases of graft rejection in DALK group than those in PKP group (OR = 0.28; 95%CI 0.15 to 0.50; p<0.001). Nevertheless the rate of graft failure was similar between DALK and PKP groups (OR = 1.05; 95%CI 0.81 to 1.36; p = 0.73). There were no significant differences in the secondary outcomes of SE (p = 0.70), astigmatism (p = 0.14) and CCT (p = 0.58) between DALK and PKP groups. And ECC in DALK group was significantly higher than PKP group (p<0.001). The postoperative complications, high intraocular pressure (high-IOP) and cataract were analyzed, fewer cases of complications occurred in DALK group than those in PKP group (high-IOP, OR 0.22, 95% CI 0.11–0.44, P<0.001) (cataract, OR 0.22; 95% CI 0.08–0.61, P = 0.004). And no cases of expulsive hemorrhage and endophthalmitis were reported.

**Conclusion:**

The visual outcomes for DALK were not equivalent to PKP. The rate of graft failure was similar between DALK and PKP. Fewer postoperative complications occurred in DALK group, indicating that compared with PKP, DALK has lower efficacy but higher safety.

## Introduction

Keratoconus is a degenerative, ectatic corneal disease with central or paracentral corneal thinning, which exhibits progressive corneal steeping and protrusion that results in increasing regular and thereafter irregular astigmatism. In case of end stage, this may lead to corneal scarring, corneal hydrops and loss of corrected distance visual acuity. The etiology and pathogenesis of the progressive disease are still not fully understood. The reported incidence ranges from 1.3 to 25 per 100,000 per year across different population, and a prevalence of 8.8–229 per 100,000 [1,2]. The past two decades, in particular, have seen exciting new developments promising to alter the natural history of keratoconus in a favorable way for the first time. Advanced treatment modalities such as newer contact lens designs, collagen crosslinking, intracorneal ring segments, photorefractive keratectomy, and phakic intraocular lenses occurred these years [3]. In the advanced stage, keratoconus may further develop into the complication stage with spontaneous Descemet’s membrane (DM) tears causing highly acute stromal edema, and even the occasional occurrence of perforation [4]. Ultimately, corneal transplantation becomes the only feasible therapeutic approach for keratoconus and about 15% to 20% of affected individuals may require a corneal transplant [5]. Penetrating keratoplasty (PKP) has been considered as the gold standard for the treatment of advanced keratoconus for decades of years owing to the safety and good visual acuity outcomes [6,7]. However, full-thickness replacement of the cornea is often associated with a risk of immune-mediated endothelial rejection, endothelial cell loss and complications such as expulsive hemorrhage and endophthalmitis [8,9,10,11]. Over the last 15 years, deep anterior lamellar keratoplasty (DALK) has become an alternative procedure to PKP by replacing the anterior portion of the diseased cornea without removing the Descemet’s membrane (DM) and endothelium, thus reducing the risk of endothelial graft rejection [12,13]. Various new techniques including hydrodissection and big-bubble technique have been introduced to DALK in an attempt to create a smooth interface and reduce interface scarring and refractive irregularities [14,15,16]. Keenan TD et al [17] reported that the annual number of corneal graft operations for Keratoconus in United Kingdom increased from 514 to 608 (1999–2000 to 2005–2006) and then decreased to about 550 (2006–2007 to 2008–2009). The number of patients undergoing PKP decreased from 88.1% to 57.1%, compared with an increase from 8.8% to 40.1% for DALK (1999–2000 to 2008–2009).

As most patients with progressive keratoconus are young, they have higher requirements on the visual acuity outcome and graft survival [18]. But as studies on DALK and PKP in recent years have reported inconsistent results in relatively small numbers of patients, the present study compared the surgical outcomes through literature retrieval in an attempt to explore the advantages and disadvantages of DALK and PKP with respect to visual acuity, refractive error and graft survival, hoping that this meta-analysis could provide quantitative and high-level evidence to help guide the clinical treatment of keratoconus.

## Materials and Methods

### 1. Definition of outcome measures

The results of visual acuity were analyzed by the rate of final postoperative best corrected visual acuity (BCVA) of patients with spectacles or contact lenses (or unaided when not available) beyond 6/12 and 6/6. Mean postoperative logarithms of minimal angle resolution as the best spectacle-corrected visual acuity (logMAR BCVA) was used to calculate visual acuity in different time points. The results of refractor error were evaluated by spherical equivalent (SE) and astigmatism. The postoperative safety was evaluated by endothelial cell count (ECC), central corneal thickness (CCT) and graft rejection. Complications were evaluated by the rate of secondary high-IOP and cataract.

### 2. Search Strategy

The meta-analysis was conducted according to the PRISMA guidelines [19] by searching the following databases: Pubmed, EMBASE, Cochrane Library, Web of science till October 1, 2014 using the Medical Subject Heading (MeSH) keywords: “deep anterior lamellar keratoplasty”, “penetrating keratoplasty”, “keratoconus”, “corneal transplantation”, “corneal grafting”, “lamellar keratoplasty”, and “corneal disease”. The references of related articles were retrieved for additional publications. The language was restricted to English.

### 3. Inclusion and Trials Selection

Studies were selected if they fulfilled the following criteria: (1) controlled clinical trials, including retrospective studies, cohort studies and prospective studies such as randomized controlled trials (RCTs), (2) confirmed diagnosis of keratoconus, excluding patients who had undergone repeated DALK or PKP, and corneal transplantation in combination with other surgeries such as cataract, (3) studies that reported the follow-up results beyond 6 months concerning DALK and PKP treatment for keratoconus, (4) studies offering at least one of the outcomes of interest.

### 4. Data Extraction

Data were extracted by two reviewers (H. Liu and B. Li) independently. Disagreement was resolved with the third participator (YH. Chen) by discussion. A customized form including the following items was used for data extraction: (1) study characteristics, including the first author, published year, country of study, and sample size; (2) patient characteristics, including the mean age, sex, follow-up period and withdrawals to follow up; (3) study design, including the type of study (RCTs, cohort or prospective studies); (4) main outcome measures including BCVA, LogMAR, graft rejection, graft failure; secondary outcome measures including SE, astigmatism, CCT, and ECC; postoperative complications including the risk of secondary glaucoma and cataract.

### 5. Assessment of methodology quality

The quality of included studies was assessed using the US Preventive Services Task Force grading system [20], Downs and Black quality assessment method [21] and the Newcastle-Ottawa Scale (NOS) [22]. The Downs and Black Scale containing a list of 27 criteria for evaluating external validity, internal validity-bias, selection bias and power of included studies was eligible for both RCTs and non-RCTs, where higher scores indicate higher qualities in the form of four ranges: ≤14, 15–19, 20–25 and 26–28. The NOS was only used to evaluate non-RCTs and consisted of selection, comparability and outcomes or exposure for cohort or retrospective studies. The maximum was 4* for selection, 2* for comparability, and 3* for outcome or exposure. The total score ranged from 0*(bad) to 9*(good), and studies with ≥6* were considered having a relatively high quality.

### 6. Statistical Analysis

Quantitative data of the outcome parameters were analyzed using the Cochrane Review Manager (RevMan) version 5.0 software program. 95% confidence interval (CI) was calculated for each study, and the weighted mean difference (WMD) was measured with the 95% CI for continuous variables, and the odds ratio (OR) was measured with the 95% CI for dichotomous variables. The Cochran Q-Statistics chi-square test and inconsistency index (I-squared, *I* ^2^) were applied to identify statistical heterogeneities. If statistical heterogeneity (*P*<0.10 or *I* ^2^>50%) was identified, random effects meta-analysis was used; if not, a fixed-effects model was used [23]. A funnel plot was also constructed to investigate the potential publication bias influencing the analysis.

## Results

### 1. Literature search

**Fig 1:**
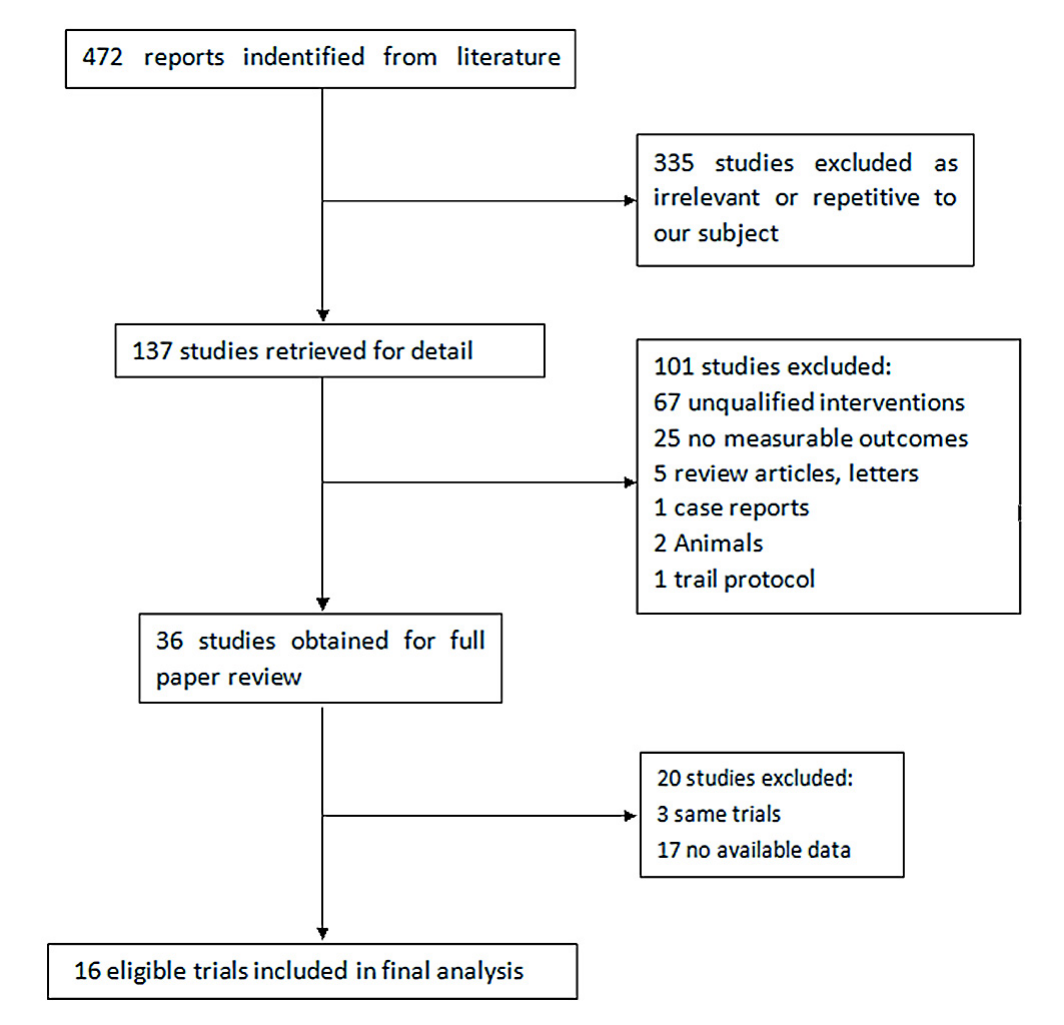
Results of the literature search strategy.

A total of 472 articles (118 from Pubmed, 9 from Cochrane, 142 from EMBASE, and 202 from web of science) were initially identified, from which we excluded those that were not qualified. Subsequently, 36 articles with full texts were assessed for eligibility. Three articles were from the same trials and 20 articles failed to provide data available. Eventually, 16 studies [24–39] published from 2004 till 2014 were included in this meta-analysis (Fig. 1).

### 2. Baseline characteristics of the included studies

The 16 included studies included 14 retrospective studies, one cohort study and one RCT, involving a total of 6625 eyes, including 1185 in DALK group and 5440 in PKP group. Ten of the 15 studies contained more than 20 eyes in both control and case groups, with a follow-up duration of more than 12 months. The study design and baseline characteristics of the qualified studies are summarized in Table 1.

**Table 1:** Characteristics of eligible studies.

			Age		Sex(Male: Female)		No. of eyes		Left: Right		Follow-up(months)	
Study	Country	Study design	DALK	PKP	DALK	PKP	DALK	PKP	DALK	PKP	DALK	PKP
Wanson.SL 2004	Britain	retrospective	32.6±10.8	33.9±13.1	17:8	14:08	25	22	12:13	9:13	28	55
Funnell.CL 2006	Britain	cohort	32±11	22±8.5	9:11	6:14	18	20	NA	NA	12	12
Irit.B 2008	Canada	retrospective	32.5±13.0	42.2±15.4	11:5	19:14	17	20	8:9	17:16	17.0±10.9	53.2±44.3
Han.DC 2009	Singpore	retrospective	30.2±10.4	26.6±9.3	NA	NA	25	100	NA	NA	16.0±10.3	28.4±11.8
Jones.MN 2009	Britain	retrospective	NA	NA	NA	NA	455	1917	NA	NA	60	60
Javadi.MA 2010	Iran	RCT	26.9±7.9	30.9±10.3	29:13	28:7	42	35	NA	NA	22.0±7.9	24.6±3.5
Cohen.CW 2010	U.S.A	retrospective	35.4±14.4	45.5±13.1	21:9	8:3	11	30	NA	NA	22.5±2.5	21.9±3.7
Kim.KH 2011	Korea	retrospective	25.3±7.3	26.2±9.8	17:2	25:13	19	38	12:7	25:13	22.6	51.7
Mashor.RS 2011	Canada	retrospective	33.4±10.8	34.6±13.9	10:8	11:7	18	18	NA	NA	17.83	11.3
Kubaloglu.A 2012	Turkey	retrospective	34.5±7.3	36.3±8.3	NA	NA	44	30	NA	NA	24	24
Amayen.AF 2012	Egypt	retrospective	24.3±6.4	26.5±8.4	33:14	24:6	47	30	NA	NA	24	24
Akdemir.MO 2012	Turkey	retrospective	29.7±5.0	28.7±3.5	14:16	16:14	30	30	NA	NA	12.6±3.7	12.6±3.7
Oh.BL 2013	Korea	retrospective	28.1±11.7	27.4±5.0	7:4	3:02	11	5	7:4	5:4	30±17	45±20
Zhang.YM 2013	China	retrospective	20.6±6.8	21.9±9.9	55:20	45:7	75	52	NA	NA	46.9±28.0	60.2±34.6
Macintyre.R 2014	Australia	retrospective	29.2±7.8	32.3±9.1	19:12	22:20	31	42	NA	NA	51.8	53.7
Coster.DJ 2014	Australia	retrospective	32±22.8	59±24.3	NA	NA	317	3051	NA	NA	NA	NA

### 3. Quality assessment

Of the 16 included studies, 7 were conducted in Asia, 3 in North America, 3 in Europe, one in Africa, and two in Australia. For the Downs and Blacks score, the scores of all studies were over 14, and the score of the RCT was 21. For NOS, 11 of the 15 non-RCTs had scores ≥6*, and the lowest score was 5* (Table 2).

**Table 2:** Description of the characteristics of the included trials.

			Study Quality				
				NOS Scale			
First Author, Years	Country	Trial Types	Downs and Black Scales	Selection	Comparability	Expose	Total Score
Watson.SL2004	Britain	retrospective	14	***	**	**	*******
Funnell.CL2006	Britain	cohort	16	***	**	**	*******
Irit.B 2008	Canada	retrospective	16	**	*	**	*****
Han.DC^]^ 2009	Singapore	retrospective	14	***	0	**	*****
Jones.MN 2009	Britain	retrospective	15	**	*	**	*****
Javadi.MA 2010	Iran	RCT	21	—	—	—	—
Cohen.CW 2010	U.S.A	retrospective	16	***	*	**	******
Kim.KH 2011	Korea	retrospective	15	***	*	**	******
Mashor.RS 2011	Canada	retrospective	15	***	*	**	******
Kubaloglu.A 2012	Turkey	retrospective	16	***	**	**	*******
Amayem.AF 2012	Egypt	retrospective	14	***	**	**	*******
Akdemir.MO 2012	Turkey	retrospective	16	***	**	**	*******
Oh.BL 2013	Korea	retrospective	16	***	*	**	******
ZHANG.YM 2013	China	retrospective	14	***	*	*	*****
Macintyre.R 2014	Australia	retrospective	15	***	*	**	******
Coster.DJ 2014	Australia	retrospective	15	***	*	**	******

— = no data provided; RCT = randomized controlled trials.

### 4. Primary outcome measures

**Fig 2:**
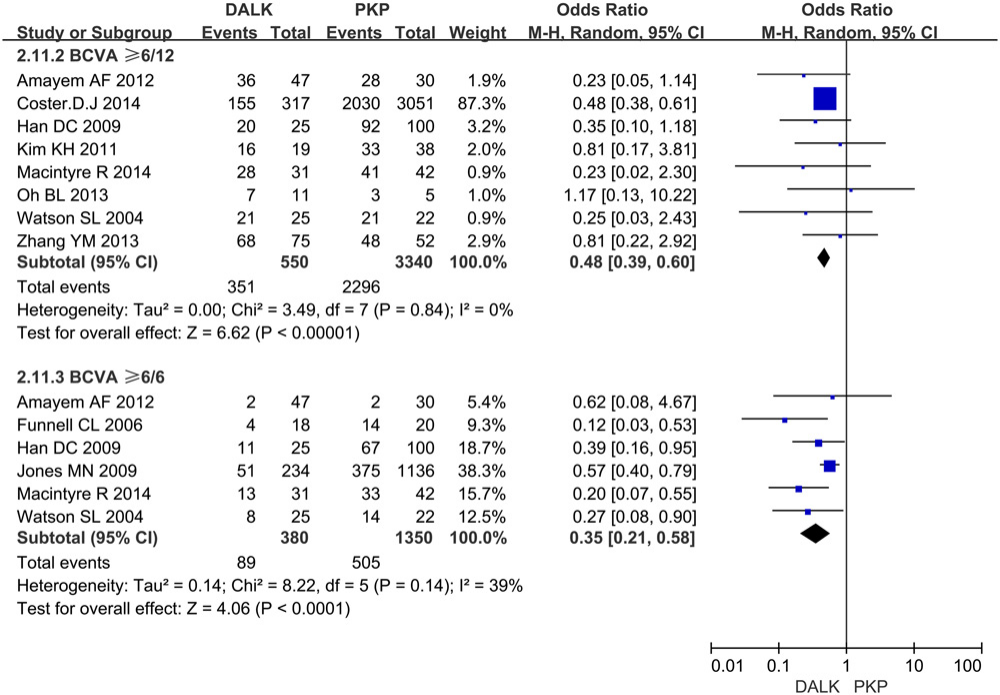
Forest plot comparing the rate of patients whose postoperative BCVA beyond 6/12 or 6/6 between DALK and PK. CI = confidence interval; SD = standard deviation; DALK = deep anterior lamellar keratoplasty; PK = penetrating keratoplasty.

**Fig 3:**
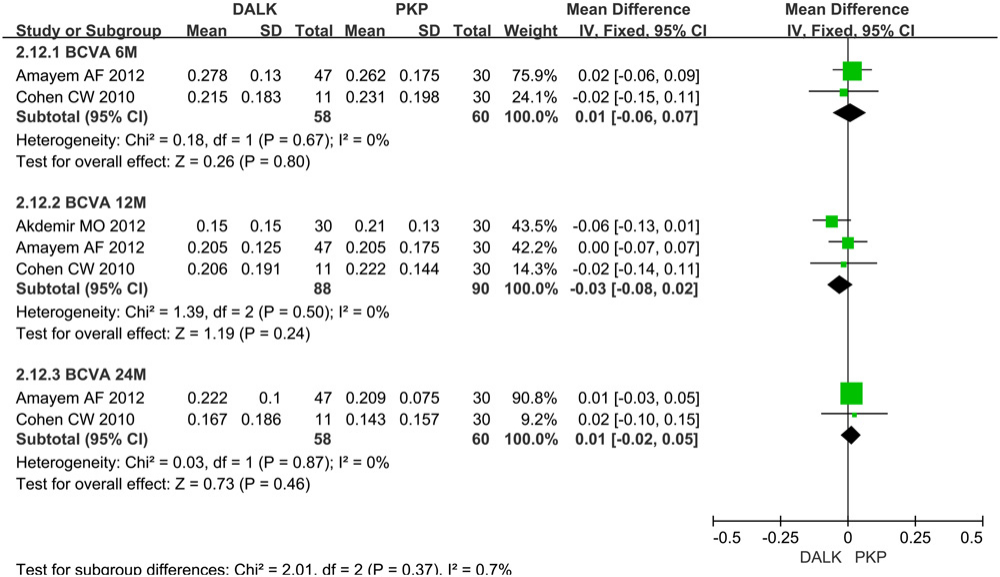
Forest plot comparing the mean LogMAR BCVA between DALK and PK in different time points. CI = confidence interval; SD = standard deviation; DALK = deep anterior lamellar keratoplasty; PK = penetrating keratoplasty.

**4.1 Visual Acuity**. Eight studies reported the number of patients whose postoperative BCVA ≥ 6/12. And 6 studies reported the number of patients whose postoperative BCVA ≥ 6/6. The synthesis of their dichotomous data showed that the rate of patients whose final postoperative BCVA beyond 6/12 or 6/6 was significantly better for PKP than that for DALK (OR = 0.48; 95%CI 0.39 to 0.60; p<0.001, OR = 0.35; 95%CI 0.21 to 0.58; p<0.001) (Fig. 2). There were 3 studies reported LogMAR BCVA in different time points. No significant difference between DALK group and PKP group was found in the follow-up of 6 month (WMD = 0.01; 95%CI -0.06 to 0.07; p = 0.80), 12 month (WMD = -0.03; 95%CI -0.08 to 0.02; p = 0.24) and 24 month (WMD = 0.01; 95%CI -0.02 to 0.05; p = 0.46) (Fig. 3).

**Fig 4:**
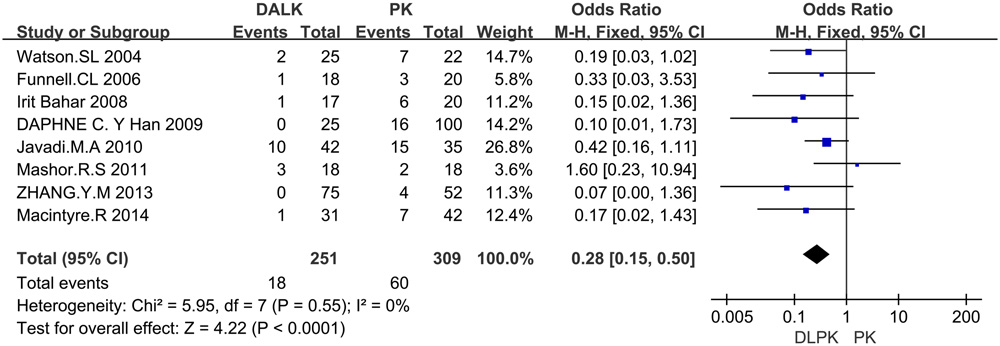
Forest plot comparing the rate of graft rejection between DALK and PK. CI = confidence interval; SD = standard deviation; DALK = deep anterior lamellar keratoplasty; PK = penetrating keratoplasty.

**4.2 Graft Rejection**. There were 8 studies reporting graft rejection, showing that graft rejection rate was significantly higher in PK group than that in DALK group (OR = 0.28; 95%CI 0.15 to 0.50; p<0.001) (Fig. 4).

**Fig 5:**
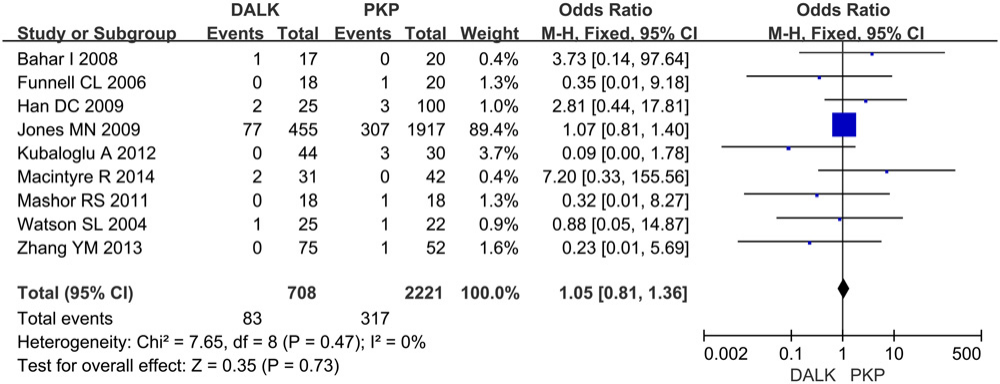
Forest plot comparing the rate of graft failure between DALK and PK. CI = confidence interval; SD = standard deviation; DALK = deep anterior lamellar keratoplasty; PK = penetrating keratoplasty.

**4.3 Graft Failure**. Nine studies reporting graft failure were included. After analyzing the data, we didn’t find significant difference between DALK group and PKP group for the rate of graft failure (OR = 1.05; 95%CI 0.81 to 1.36; p = 0.73) (Fig. 5).

### 5. Secondary outcome measures

**5.1 Spherical Equivalent**. Eleven studies reported the final refractive spherical equivalent and used the random effects model to analyze the data for heterogeneity (*I* ^2^ = 91%). There was no statistically significant difference between the two groups (WMD = -0.22; 95%CI -1.35 to 0.91; p = 0.70)

**5.2 Astigmatism**. Eleven trials reported the postoperative mean astigmatism using the random effects model (*I* ^2^ = 77%). The PK group tended to have a higher astigmatism than DALK group, but the difference was not statistically significant (WMD = 0.51; 95%CI -0.16 to 1.19; p = 0.14)

**5.3 Central corneal thickness**. Four studies scored central corneal thickness using the random effects model (*I* ^2^ = 58%), showing no significant difference between DALK and PKP groups (WMD = 5.89; 95%CI -15.19 to 26.98; p = 0.58).

**5.4 Endothelial cell count**. After analyzing 3 included studies, the result showed the ECC in DALK and PKP group patients during the follow-up periods of more than 6-months, showing that the mean ECC in PKP group was significantly lower than that in DALK group (WMD = 926.53; 95%CI 772.46 to 1080.60; p<0.001).

### 6. Complications

The operative high intraocular pressure (IOP) and cataract were analyzed. The results demonstrated significantly differences between DALK and PK groups. The rate of postoperative high-IOP and cataract in PKP group was higher than that in DALK group (high-IOP, OR 0.22; 95% CI 0.11–0.44; p<0.001) (cataract, OR 0.22; 95% CI 0.08–0.61; p = 0.004). Meanwhile no complications of expulsive hemorrhage and endophthalmitis were reported after analyzing the included 16 articles. There were 5 articles reported 5 cases required conversion to PKP. And the rate of conversion was about 2.96%.

Primary outcomes, secondary outcomes and complications are listed in Table 3.

**Table 3:** Description of the primary outcomes, secondary outcomes and postoperative complications of meta-analysis.

outcomes	No.of studies	No. of eyes		Overall effect		Heterogeneity	p Value
		DALK	PKP	WMD/OR(95%CI)	p Value	I^2^,%	
BCVA≥6/12	8	500	3340	0.48(0.38, 0.59)	0.84	0%	<0.001
BCVA≥6/6	6	380	1350	0.46(0.35, 0.61)	0.14	39%	<0.001
Log MAR 6M	2	58	60	0.01(-0.06, 0.07)	0.67	0%	0.80
Log MAR 12M	3	88	90	-0.03(-0.08, 0.02)	0.50	0%	0.24
Log MAR 24M	2	58	60	0.01(-0.02, 0.05)	0.87	0%	0.46
Graft rejection	8	251	309	0.28(0.15,0.50)	0.55	0%	<0.001
Graft Failure	9	708	2221	1.05(0.81, 1.36)	0.47	0%	0.73
SE	11	738	2187	−0.22(-1.35, 0.91)	<0.001	91%	0.70
Astigmatism	11	314	312	0.51(-0.16, 1.19)	<0.001	77%	0.14
CCT	4	108	100	5.89(-15.19,26.98)	0.07	58%	0.58
ECC	3	72	55	926.53(772.46, 1080.60)	0.29	19%	<0.001
High-IOP	6	227	255	0.22(0.11, 0.44)	0.12	45%	<0.001
Cataract	4	142	224	0.22(0.08, 0.61)	0.25	27%	0.004

### 7. Heterogeneity and publication bias

Some outcomes displayed great heterogeneity as shown in Table 3. The heterogeneity of SE and astigmatism was significant, and dropping eligible studies by hand and Meta regression have not provided good results. However, the studies included in the analysis of CCT were also heterogeneous, and the heterogeneity of CCT was not as significant as before after dropping a study ^[36]^ (I^2^ = 32%, p = 0.86). No significant publication bias was demonstrated in the funnel plot.

## Discussion

The results of this meta-analysis show that patients who undergoing DALK had a poorer BCVA compared with PKP (p<0.001). Nevertheless comparable LogMAR BCVA was found in the follow-up of 6 month, 12 month and 24 month between DALK group and PKP group (p>0.05). The rate of graft rejection in DALK group was significantly lower than that in PKP group (p<0.001), but the rate of graft failure was similar (p>0.05). Preservation of the corneal endothelium in DALK group was much better than that in PKP group (p<0.001). Less complications occurred in DALK group compared with PKP group (p<0.001). This meta-analysis also shows that there was no significant difference in SE, CCT and astigmatism between DALK and PKP groups (p>0.05), indicating that compared with PKP, DALK has lower efficacy but higher safety.

Penetrating Keratoplasty, a procedure consisting of full-thickness replacement of the cornea, has been the dominant procedure and successfully caters to most causes of corneal blindness for half a century [40]. DALK was initially introduced by Archila in 1985 [41]. For avoidance of intraocular tissue damage, postoperative endothelial rejection and complications of open-sky procedure during surgery, DALK has become an alternative surgical procedure to PKP in the treatment of a variety of keratonosus such as keratoconus, trauma, degenerations and dystrophies [33]. Due to remaining stromal layer and having hydrops between host-donor interface, interface haze and residual scarring in DALK, earlier studies showed PKP had better outcomes of acuity vision [41,42]. These suboptimal visual outcomes were mainly attributed to the earlier less advanced techniques such as manual lamellar dissection [26,43]. New DM-baring techniques such as Anwar that remove and replace total weak keratoconic stroma seem to provide more satisfied visual results [14,44,45].

Many studies have compared the outcomes of DALK and PK, reporting inconsistent results with respect to visual acuity [43,46,47]. Our meta-analysis indicates PKP achieved a better visual acuity by calculating BCVA. However comparable visual outcomes were obtained between PKP and DALK group by calculating LogMAR BCVA in different time points. We believe the results were not incompatible. The mean BCVA were comparable, meanwhile the rate of postoperative BCVA beyond 6/12 and 6/6 was higher for patients undergoing PKP. Several studies [41,42,43] have documented that DALK is inferior compared to PKP. Some other studies [48,49,50] indicated that the visual outcomes after DALK were comparable with those after PKP. The difference may be attributed to the different surgical procedures and technical skills leading to irregularity at host-donor interface which limiting vision after DALK and lack of various methods to evaluate visual outcomes.

Some previous studies noted a trend toward higher myopia in DALK patients [24,28], but recent studies [30,51] presented that the postoperative occurrence of myopia was similar between DALK and PK patients, which is consistent with our analysis. Three factors were hypothesized as contributing to this result: residual stromal thickness, corneal curvature and axial length. According to our data analysis, the mean CCT between the two groups was similar and there was no significant difference. This phenomenon probably is mainly due to new techniques of DALK, which leads to less hydrops and residual stromal thickness [14,15]. In addition, an oversized donor button may result in higher corneal curvature and longer axial length, which attribute to higher myopic refractive outcomes [36,52]. Watson et al and Shimazaki et al [24,53] reported no significantly different astigmatism between the two groups, which is consistent with our analysis. The corneal endothelium is preserved after DALK, thus minimizing the risk of endothelial rejection. Sarnicola et al [54] reported no progressive endothelial cell loss in postoperative DALK patients during a 6-month follow-up period. Five-year long-term data showed that the mean 5-year endothelial cell loss was 22.3% in the DALK group and 50.1% in PKP group, which is similar to the finding of Kubaloglu A et al [35]. Although the mean ECC in PKP group was reduced more than that in DALK group, but we found there was no statistically significant difference between the mean CCT of the two groups. The similar results were reported by Kettesy B [55], who found that there was no correlation between ECC and corneal thickness. Kus MM [56] proved that even if ECC decreased significantly, as long as an acceptable density was kept, the remaining cells compensated the loss well and corneal thickness remains normal. Extrapolation from observed endothelial cell loss rates predicts a median graft survival of 49 years for DALK compared with 17 years for PKP [57].

Although PKP is an effective treatment for keratoconus, its disadvantages cannot be ignored. The transplanted cornea is at a risk of rejection and endothelial loss, for the sake of PKP sacrificing the healthy endothelium of the host cornea. Ing JJ et al [58] reported that the rate of corneal endothelial loss after PKP was several times higher than that in a normal population. In contrast, the major advantage of DALK *vs*. PKP is the low rate of endothelial cell loss and graft rejection, which is the right goal expected by surgeons all over the world. As the DM and endothelium of the patient are preserved, the corneal endothelial density in DALK is significantly higher than that in PKP, and the occurrence of allograft rejection in DALK patients is much lower [11,50]. These findings are consistent with our meta-analysis. Our results showed that the rate of graft failure was similar between DALK and PKP groups. Reinhart WJ [59] has reported a similar results. It was indicated that the major reasons of graft failure for DALK were host-donor interface haze and stromal scarring. For PKP, the major reason was endothelial rejection [46].

DALK avoids an open-sky procedure and reduces related complications such as cataract and glaucoma [26,29]. Although DALK eliminates the risk of endothelial rejection, stromal and epithelial rejections were similar between DALK and PKP [31,42]. However, stromal rejection is usually mild and can be attenuated quickly by topical steroids [27,42]. As rejection is relatively rare and mild in DALK, it requires a shorter postoperative steroid regimen, which reducing the risk of complications such as cataract and glaucoma. In addition, the suture can be removed earlier, which beneficial to postoperative rehabilitation [26,60]. After analyzing the conversion of DALK, we found 3 cases were due to perforation, 1 case was due to ectasia at the graft-host junction and 1 case was due to polyplopia. There are still technical difficulties for DALK, and it depends on the skill and technique of individual surgeons. Thus the surgical procedure may be prolonged and cause complications associated with the graft-host interface such as microperforation and secondary anterior chamber [27].

This meta-analysis still has some limitations. First, most of the studies included in this meta-analysis were retrospective rather than prospective clinical studies, which may affect the reliability of the results due to possible selection bias, known or unknown confounding bias and reporting bias. In addition, some parameters had relatively large heterogeneity. The heterogeneity of SE and astigmatism were not explained due to different surgical techniques, different methods of measurement or different follow-up periods in different trials. However, we still believe that the results of this meta-analysis are useful because it includes a relative large number of studies and cases that provide a strong power and the consonance of previous results and sensitivity analysis.

In conclusion, the visual outcomes of BCVA for DALK were better than those for PKP. In terms of refractor error, the outcomes of DALK and PKP for keratoconus are equivalent. DALK showed its advantages in preservation of the corneal endothelium and the reduced risk of graft rejection and complications. DALK seems to be an alternative therapy for the treatment of keratoconus with lower efficacy but higher safety.

## Supporting Information

S1 PRISMA Checklist PRISMA Checklist of items reporting meta-analysis. (DOC)
